# Effects of Exercise and Enzyme Therapy in Early Occupational Carpal Tunnel Syndrome: A Preliminary Study

**DOI:** 10.1155/2019/8720493

**Published:** 2019-01-23

**Authors:** Věra Žídková, Marie Nakládalová, Ladislav Štěpánek

**Affiliations:** ^1^Department of Occupational Medicine, University Hospital Olomouc and Faculty of Medicine and Dentistry, Palacký University Olomouc, Czech Republic; ^2^General Practitioner, Baška, Czech Republic

## Abstract

**Objectives:**

Occupational carpal tunnel syndrome (CTS) due to upper extremity overuse has in recent years been the most commonly recognized occupational disease in the Czech Republic and its prevalence has steadily increased. This pilot observation aimed to assess the effects of exercise techniques and oral enzyme therapy in automotive plant workers with early CTS.

**Patients and Methods:**

The observation comprised automotive plant assembly line workers in whom nerve conduction study revealed incipient CTS. The subjects were divided into three groups: a group practicing exercise techniques (exercising; N=15), a group receiving oral enzyme therapy (N=16), and a group of controls (N=14). Subjects in the control group were only observed without any specific intervention, which is a common procedure in incipient CTS. Throughout 9-week observation, the workers did their jobs. Prior to and after that period, the workers' CTS-related symptoms were ascertained through structured interviews with a physician and the following median nerve parameters were measured: sensory conduction velocity (SCV) and distal motor latency (DML).

**Results:**

In both the exercise and enzyme therapy groups, statistically significant decreases in the total score for symptoms were achieved (p<0.0001), unlike controls. On final examination, both treated groups demonstrated significantly increased SCV as compared with the initial values (p=0.00013 and p<0.0001, respectively); in controls, the mean SCV did not significantly change. Similarly, a statistically significant shortening of DML was noted in the enzyme therapy group (p=0.008).

**Conclusion:**

The results showed the efficiency of both exercise and oral enzyme therapy in incipient CTS. These methods may be recommended for preventing more severe forms of CTS.

## 1. Introduction

Repetitive strain injury has been recognized as an occupational disease in the Czech Republic since 1975 [1]. While the incidence of the other occupational diseases has generally decreased, the trend is opposite for this group of conditions [2]. Occupational carpal tunnel syndrome (CTS) due to upper extremity overuse has been the most common occupational disease in the Czech Republic for more than 10 years. This condition has been most frequently observed in the automotive industry in the last three years [3, 4].

This is also true for the studied plant where automobile parts are assembled, with CTS being the most common health problem in workers exposed to upper extremity overuse. Despite the fact that several targeted technological and organizational preventive measures were put in place in the plant (e.g., improved workplace ergonomics or directed rotation of workers between various operations), the health problem still persists [5].

Therefore, its prevention and therapy are a hot issue. CTS management is both surgical (especially in advanced conditions) and conservative, including splints keeping the wrist at a neutral position, especially at night, topical corticosteroids, nonsteroidal anti-inflammatory drugs, therapeutic ultrasound, job change, yoga, acupuncture, oral enzyme therapy, or kinesiotherapy [6–10]. However, uniform standard evidence-based guidelines for treating CTS are still lacking [11].

The study is aimed at assessing the effect of exercise techniques and oral enzyme therapy in the incipient forms of median nerve damage at the wrist in workers exposed to upper extremity overuse in an automotive plant.

## 2. Materials and Methods

### 2.1. Subjects and Study Protocol

The prospective pilot observation comprised automotive plant assembly line workers in whom, during annual periodic preventive occupational health examination, nerve conduction study (NCS) revealed incipient median neuropathy at the wrist, who were willing to participate in the project, and in whom the following states were not found out: previously injected corticosteroids for CTS, inflammatory joint disease, a history of trauma to the affected hand, surgery for CTS, pregnancy, polyneuropathy, and other relevant states. These forms of slightly abnormal NCS findings are only a matter for increased observation without any targeted intervention. Informed consent was obtained from all participants after explaining all information regarding the project to them.

All workers (N=45) were exposed to the risk factor local muscular strain to the upper extremities [12]. Their work tasks, including installation of engines, gearboxes, superchargers, shock absorbers, and other parts, were performed while standing. Workers mostly handled small parts or loads of up to 10 kg; only rarely and irregularly, pneumatic torque wrenches were used.

Workers were offered three possible ways of participation based on their preferences expressed during the initial medical examination: in a group practicing exercising, in a group receiving oral enzyme therapy, and in a group of controls. The group composition by gender and age is shown in Table 1. The subjects were gradually enrolled between mid-2014 and mid-2016; throughout the study period, the workers did their jobs.

Initially and after nine weeks, workers were asked about CTS-related symptoms and underwent NCS of the median nerve. For keeping compliance, the exercise and enzyme therapy groups had additional medical appointments after three and six weeks, during which they were reeducated and motivated to exercise and use their medication regularly. Also tolerability of enzyme therapy was assessed.

### 2.2. Data Collection

During a structured interview, the worker's symptoms were recorded by a physician, for each hand separately. The questions were focused on the type, intensity, duration, and frequency of symptoms. Also noted during the interview were difficulties performing common daily activities caused by impaired fine hand motor skills (e.g., manipulating coins, buttons, or zippers, using mobile phones, and opening plastic bottles). For statistical analysis, symptoms were assigned points based on their severity (see Table 2). Provocative tests (Tinel's, Phalen's, and DelPino's) were also included and assessed as a part of the point score.

In our study, the core objective method for assessing the prevalence and severity of CTS was a NCS test of the median nerve at the wrist. The main studied parameters were distal motor latency (DML) and sensory conduction velocity (SCV). All NCS tests were carried out by a single neurologist in accordance with standard methods. That is, SCV was determined to the 2nd or 3rd finger at 14 cm and DML of the median nerve at 8 cm at an angle [13].

Normal conduction parameters of the median nerve were SCV ≥ 50.00 m/s and, at the same time, DML < 4.20 ms. In the study, moderate neuropathy was defined as SCV 38.10–40.00 m/s and, at the same time, DML 4.90–5.29 ms. These moderate forms of neuropathy were the reason for relocating the worker to another job because of the risk of developing occupational disease. Cases neither reaching this grade nor showing normal values were labeled as incipient median neuropathy and included in the study. Cases having SCV ≤ 38.0 m/s and, at the same time, DML≥ 5.30 ms, together with relevant clinical findings, are considered as having an occupational disease in the Czech Republic. For clarity purposes, motor and sensory conduction were assessed separately; however, there were some workers with combined sensorimotor lesions (Table 1).

### 2.3. Exercise Techniques

Individuals in the exercise group performed three simple techniques with neuromobilization elements at least once daily throughout the study period [14]. They were given explanation about the principle and aim of the exercise and were adequately educated about how to perform the techniques. They received both a printed leaflet with pictures of the techniques and an audiovisual recording showing the techniques.

**Figure 1:**
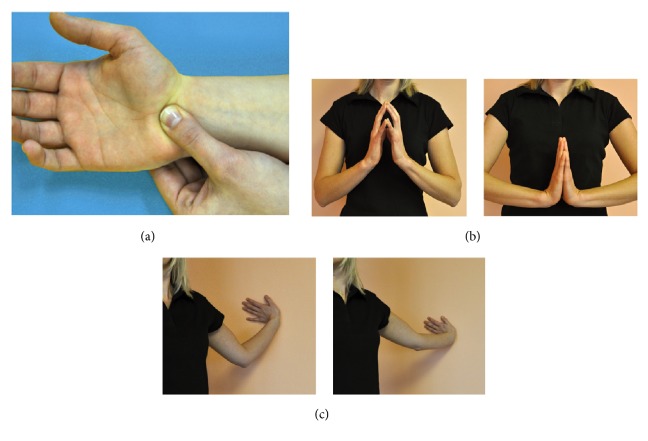
Three exercise techniques performed by the exercise group.

The first preparative technique (Figure 1(a)) focuses on soft tissue treatment, fascicular mobilization of the retinaculum flexorum. The aim of the technique is to adjust flexibility of this soft tissue with a deep push and pull in order to prevent its fibrosis. Through the preserved flexibility of the area, more space is gained for the median nerve. The technique is pushing and pulling from the carpal bones to the palm. The back of the wrist is stretched with the opposite thumb moving from place to place, leaving the thumb in each place for about 3 seconds. In total, the technique should be performed for about 30 seconds.

The second technique (Figure 1(b)) concentrates on the relaxation of flexor muscle groups using the “hold-relax” technique with the neurophysiological effect of postisometric relaxation. The technique requires that the person stands or sits straight. The palms are clasped in front of the chest. First, the fingers press against each other for 5 seconds and then the pressure is released for another 5 seconds. Then the palms are placed together and moved towards the abdomen. As with the third technique, minimal and maximal numbers of repetitions are set, and the actual number of repetitions is up to the individual tolerance. This technique is repeated two or three times.

The third technique (Figure 1(c)) is a neuromobilization technique, using slide and strain of the median nerve. To perform the technique, the person stands with their side to a wall with the arm stretched and the palm leaning against the wall; the hand is slightly rotated. The extended arm is bent at the elbow and extended again. Thus, the elbow joint is slowly extended from the flexed position by moving the entire trunk. The technique is performed 6 to 8 times. Additionally, if possible the head is tilted towards the opposite shoulder, which makes the technique more effective through acting on more proximal parts of the median nerve. As a result of these techniques, the nerve adapts to the change in pressure over various extreme positions.

### 2.4. Enzyme Therapy

The second group was put on a 9-week course of oral enzyme therapy with Wobenzym (MUCOS Pharma GmbH & Co. KG, Germany), an over-the-counter drug in enterosolvent tablets containing, in the administered daily dose (20 pills divided into two doses), 2000 mg pancreatin, 900 mg bromelain, 1200 mg papain, 480 mg trypsin, 20 mg chymotrypsin, 200 mg amylase, 200 mg lipase, and 1000 mg rutin. It has anti-inflammatory, antiedematous, and analgesic effects. For these properties it is used for treatment of various types of musculoskeletal disorders [15, 16]. At the beginning of the study, all workers were informed about the reasons for and effects of the therapy, way of administration, and potential adverse effects.

Tolerability of the enzyme therapy was assessed during each appointment through targeted questions. Five cases of adverse effects were reported. These were previously known common side effects affecting the digestive system (changed stool consistency, abdominal discomfort) of mild intensity in two women and three men. The difficulties lasted for 3 to 20 days, spontaneously resolved, and were not a reason to discontinue the medication.

### 2.5. Statistical Analysis

Statistical analysis was performed with IBM SPSS Statistics Version 21 Release 21.0.0.0. Normality of initial values in the groups was assessed with the Kolmogorov–Smirnov test which ruled out statistically significant differences in age and gender distribution between the groups. SCV was evaluated using ANOVA repeated measures. The significance of changes in DML was analyzed with the paired samples t-test. The significance of changes in the final score of symptoms as compared with the initial score was tested with the Wilcoxon signed-rank test.

## 3. Results

In all observed groups, isolated sensory or motor conduction lesions in one or both extremities were detected as well as combined sensorimotor lesions. Motor conduction lesions and combined sensorimotor lesions indicate more severe states. In all extremities with symptoms, abnormal NCS findings were also noted; the only exception was one case in the exercise group. On the other hand, there were several asymptomatic cases with positive NCS findings. These included two cases in the enzyme therapy group and five in the control group (Table 1). The effects of interventions were evaluated in the three following parameters.

### 3.1. Symptoms

The analysis included all extremities with symptoms on initial examination, namely, 22 extremities in the exercise group, 24 extremities in the enzyme therapy group, and 13 extremities in the control group. In both the exercise and enzyme therapy groups, statistically significant decreases in the total score were achieved (p<0.001). In controls, the total score remained practically unchanged (Table 3).

Difficulties performing aforementioned common daily activities were not frequent. They were noticed in 7 extremities of the enzyme therapy group (5 of them stated as only occasionally), in 4 extremities of the exercise group (2 of them only occasionally), and in 3 extremities of the control group. Provocative tests were positive only in 5 extremities; 3 of them were in the exercise group, 2 in the control group, and none in the enzyme therapy group.

### 3.2. Sensory Conduction (SCV)

The statistical evaluation included 48 extremities with impairment of the sensory conduction in the median nerve at the wrist. There was no statistically significant difference in initial SCV values between the three groups. On final examination, both the exercise and enzyme therapy groups demonstrated significantly increased SCV as compared with the initial values (p<0.001). In controls, the mean SCV did not significantly change compared to the initial value (p=0.660), as seen from Table 4. There was no significant difference in the intervention effect between the exercise and enzyme therapy groups (p=0.4).

### 3.3. Motor Conduction (DML)

The study assessed a total of 28 extremities with impairment of the motor conduction in the median nerve. Most abnormal findings were observed in the enzyme therapy group. Distribution of DML findings in all groups and their development is shown in Table 5. Upon completion of oral enzyme therapy, a statistically significant improvement in DML compared to the initial value was noted (p=0.008). In that group, improvement was achieved in 10 out of 17 abnormal NCS findings; of those, 6 cases were in the normal range after 9 weeks. Given the small number of abnormal NCS findings of DML in the exercise and control groups, statistical analysis was not possible.

A certain minute improvement in the mean DML, SCV, and total scores for symptoms was also noted in the control group; this may be explained by the fact that, unfortunately, two control subjects did not adhere to the study protocol and took several days off work during the observation period.

## 4. Discussion

In the present study, a significant positive effect of specific exercise techniques and oral enzyme therapy on CTS manifestations was achieved. The effectiveness of applied procedures was confirmed on both symptoms and NCS parameters.

CTS is a major global health burden. In the literature, various definitions for diagnosing CTS are used; these are based on the presence of various symptoms, clinical signs, provocative test results, and electrodiagnostic abnormalities which is associated with the absence of international standards for diagnosing the condition [17]. Therefore, there are considerable differences in the incidence and prevalence rates stated in the literature, depending on the criteria used [18]. Thus, CTS therapy is a current issue; recently, it has also involved kinesiotherapy and neuromobilization techniques that are based on a series of both active and passive movements aimed at restoring normal properties of the nerve [11, 14].

Oskouei et al. compared the efficacy of neuromobilization techniques in individuals with mild to moderate CTS (20 persons and 32 CTS cases). The patients were divided into two groups. Both groups underwent standard physiotherapy (a wrist splint worn night and day, electrical nerve stimulation, and therapeutic ultrasound). Additionally, one group received two neuromobilization maneuvers. The study results showed that in both groups, symptoms such as tingling, numbness, weakness, and pain were significantly reduced. There were also improvements in the median nerve tension test and Phalen's sign (p = 0.005). However, NCS and hand function test results were significantly improved in the neuromobilization group. The authors therefore concluded that neuromobilization in combination with routine physiotherapy improved certain clinical findings more effectively than routine physiotherapy alone [19]. In our study, the benefit of neuromobilization was confirmed as well. Although the exercise techniques differ from those in our study, a neuromobilization effect on the median nerve may be similar.

Kwolek and Zwolińska reported significant improvements in the quality of sensation, range of motion at the wrist, and hand muscle strength in 61 patients not only immediately after completion of rehabilitation (therapeutic ultrasound, massage, and kinesiotherapy) but also one year later [20].

De-la-Llave-Rincon et al. carried out a prospective case series to examine the combined effect of soft tissue mobilization and neurodynamic mobilization of the entire median nerve in 18 women suffering from chronic CTS. The assessments were performed at baseline, immediately after the intervention and 1 week after completion of the therapy. The above techniques decreased the intensity of pain but failed to improve pressure pain sensitivity. The intensity of pain was noticed also in our study as a part of the symptom score reduction. Exercise techniques in this study performed with a physiotherapist are focused also on proximal parts of the median nerve. In case of our suggested exercise techniques, the proximal parts are slid in the third technique [21].

However, the effect of neuromobilization was not always observed. Heebner and Roddey studied the effect of neuromobilization added to standard therapy. Sixty CTS patients were assigned to two groups. The first group underwent standard therapy including patient education (discussion on the definition, anatomy, cause, and risk factors of CTS; stressing healthy lifestyle choices such as posture correction exercises, changing work ergonomics to reduce repetitive or sustained hand strain, limiting repetitive overuse stress, reducing prolonged wrist flexion or extension, decreasing salt intake, and not smoking), wearing neutral wrist splints at night, and performing eight tendon-gliding exercises. In addition to that, individuals in the other group were instructed to repeat a neuromobilization exercise ten times on three to five occasions a day (with the exercise being similar to the third technique in the present study). The outcomes were assessed at baseline and after one and six months, using the Arm, Shoulder, and Hand Questionnaire, the Brigham and Woman's Hospital Carpal Tunnel Specific Questionnaire (CTSQ), and elbow extension range of motion during a median nerve tension test. No significant differences between the groups were noted, with the only exception being improved scores on the CTSQ function scale in the first group after six months. However, the authors admit that chronicity of symptoms, poor self-reported compliance, and inadequate follow-up sessions may have adversely affected the study outcomes. The frequency of reeducation probably did not provide sufficient compliance compared to our higher frequency. Additionally, more severe forms of CTS were included in the study by Heebner and Roddey than in our observation [22].

Studies confirming the beneficial effects of enzyme therapy in musculoskeletal and extremity peripheral nerve disorders due to overuse are difficult to find in the literature. Specific problems related to upper extremity overuse and the resulting conditions (tendinitis, tenosynovitis, epicondylitis, entrapment neuropathy) leading to sick leave were addressed in a retrospective study by Zlámal showing that workers with the above-mentioned occupational exposure and conditions who underwent oral enzyme therapy in addition to obligatory therapy (rest, oral analgesics, topical analgesic, and anti-inflammatory ointments) required a significantly shorter treatment time [23].

Nakládalová et al. studied the effects of enzyme therapy in female electric motor winders with CTS. Oral enzyme therapy was shown to have beneficial effects on both the incidence and severity of median neuropathy at the wrist. The assessment was performed using both a symptom questionnaire and NCS tests [24].

In the present study, subjects themselves could select their experimental group. Therefore, the enzyme therapy group was filled earlier. The study showed that workers mostly preferred medication to exercise. On the other hand, those who learned the recommended techniques continued performing them even after the study was completed as they brought them relief.

The study outcomes suggest beneficial effects of both exercise techniques with neuromobilization and oral enzyme therapy in early CTS. There were improvements in both symptoms and NCS test results. Another benefit of the study is that after the initial education, the subjects were able to perform the techniques on their own, which saves the patient's time as well as the physiotherapist's work as sessions are not required. Moreover, patients may exercise in accordance with their needs and possibilities. We plan to continue the research by extending the sample size and assessing the effect of oral enzyme therapy combined with exercise techniques.

### 4.1. Limitations

The study has its limitations. Boston CTS questionnaire was not applied; however, there is significant similarity with the questionnaire used in our study. Relatively small sample size is another limitation. A certain limitation of the study was the fact that two control subjects did not completely adhere to the study protocol and took several days off work; on the other hand, the effect of resting was noticeable.

## 5. Conclusions

The pilot observation confirmed the efficiency of both exercise techniques and oral enzyme therapy on early stages of occupational median neuropathy at the wrist—the CTS. These methods may be recommended for improving CTS manifestations and preventing more severe forms of CTS.

## Figures and Tables

**Table 1:** Sample composition by age, gender, and initial findings.

**group**	**persons (N)**	**males (%)**	**females (%)**	**age**			**extremities with difficulties (N)**	**extremities with abnormal NCS (N)**			
				**ME**	**min.**	**max.**		**total**	**SCV**	**DML**	**SCV+DML**
**exercise**	15	12 (80.0%)	3 (20.0%)	35	24	54	22	21	16	5	0
**enzyme therapy**	16	10 (62.5%)	6 (37.5%)	31	22	59	24	26	17	17	8
**controls**	14	12 (85.7%)	2 (14.3%)	35	24	60	13	18	15	6	3

ME: median, NCS: nerve conduction study, SCV: sensory conduction velocity, DML: distal motor latency.

**Table 2:** Assessment of symptoms.

**Asking about symptoms**	**presence of symptoms/difficulties**	**points**	**hand**	
			**right**	**left**
**Type of symptoms (DAY/ NIGHT);** **intensity of symptoms:** **no – unbearable (0-5)**	pains	0–5/0–5		
	tingling, burning	0–5/0–5		
	other	0–5/0–5		

**When symptoms occur**	never	0		
	during work	1		
	at rest	1		
	at night	1		

**How long symptoms last**	never	0		
	less than 10′	1		
	10–60′	2		
	more than 60′	3		
	Permanently	4		

**DAY** **How often symptoms occur**	never	0		
	1–2x	1		
	3–5x	2		
	more than 5x	3		
	permanently	4		

**NIGHT** **How often symptoms woke him/her up **	never	0		
	1–2x	1		
	3–5x	2		
	more than 5x	3		
	permanently	4		

**Difficulties performing activities needing fine hand motor skills**	yes/no	1/0		

**Provocative tests**	Phalen/Tinel/DelPino	1/1/1		

**TOTAL**				

**Table 3:** Development in symptoms.

**extremities with symptoms in the group(N)**	**medical examination**	**total score of symptoms (mean) ± SD**	**ME**	**percentile**		**statistical significance**
				**25**	**75**	
**Exercise** **N=22**	initial	9.6±4.8	7	7	9.8	p<0.001
	final	2.0±2.8	0	0	5	

**enzyme therapy** **N=24**	initial	11.5±7.6	8	7	14.8	p<0.001
	final	5.5±6.5	6	0	8	

**controls** **N=13**	initial	11.7±4.9	9	8	14.5	p=0.161
	final	10.9±5.7	10	8	13.5	

SD: standard deviation, ME: median.

**Table 4:** Assessment of the development in sensory conduction of the median nerve.

**sensory lesions in the group (N)**	**NCS**	**mean SCV (m/s) ± SD**	**95% confidence interval for the mean**		**significance of the change**
			**lower limit**	**upper limit**	
**Exercise** **N=16**	initial	47.14±1.53	46.32	47.95	p<0.001
	final	53.11±7.57	49.07	57.14	

**enzyme therapy** **N=17**	initial	45.32±3.03	43.76	46.88	p<0.001
	final	54.75±8.44	50.41	59.09	

**controls** **N=15**	initial	45.70±2.64	44.24	47.16	p=0.660
	final	46.38±1.94	45.31	47.45	

NCS: nerve conduction study, SCV: sensory conduction velocity, SD: standard deviation.

**Table 5:** Assessment of the development in motor conduction of the median nerve.

**motor lesions in the group (N)**	**NCS**	**Mean DML (ms) ± SD**	**95% confidence interval for the mean**		**significance of the change**
			**lower limit**	**upper limit**	
**Exercise** **N=5**	initial	4.24±0.09	4.13	4.35	
	final	4.14±0.07	4.06	4.22	

**enzyme therapy** **N=17**	initial	4.44±0.28	4.3	4.58	p=0.008
	final	4.10±0.41	3.88	4.33	

**controls** **N=6**	initial	4.45±0.21	4.23	4.67	
	final	4.22±0.28	3.92	4.51	

NCS: nerve conduction study, DML: distal motor latency, SD: standard deviation.

## Data Availability

The data used to support the findings of this study are available from the corresponding author upon request.
